# Targeting RIPK1,2,3 to combat inflammation

**DOI:** 10.18632/oncotarget.6106

**Published:** 2015-10-13

**Authors:** Alex N. Bullock, Alexei Degterev

**Affiliations:** Structural Genomics Consortium, University of Oxford, Old Road Campus, Oxford, UK; Department of Developmental, Molecular & Chemical Biology, Tufts University School of Medicine, Boston, Massachusetts, USA

**Keywords:** RIPK1, RIPK2, RIPK3, inflammation, necroptosis

Receptor Interacting Protein Kinases (RIPKs) are a seven member family of human dual specificity (Ser/Thr and Tyr) kinases. Several members of this family, namely RIPK1, RIPK2, RIPK3 and RIPK7/LRRK2, have been implicated in human disease. However, besides RIPK1 and RIPK3, few commonalities have emerged with respect to the functional roles of these kinases. In this letter, we discuss recent work suggesting common themes in the cellular pathways regulated by RIPK1/3 and RIPK2 that may be relevant for the development of new therapeutic approaches against human inflammatory pathologies.

RIPK1 and RIPK3 kinase activities have been linked to the process of regulated necrotic cell death or “necroptosis”, which contributes to a variety of necrotic injuries *in vivo* (reviewed further in [1]). Furthermore, these kinases combine to form the “necrosome” complex when stimulated by Tumor Necrosis Factor alpha (TNFα). In contrast, RIPK2 mediates pro-inflammatory signaling from the bacterial peptidoglycan-sensing NOD1/2 sub-family of innate immune Pattern Recognition Receptors (PRRs) and is a putative contributor to a range of chronic inflammatory granulomatous diseases, including Inflammatory Bowel Disease (IBD) (reviewed further in [2]). However, new small molecule and genetic tools have enhanced our understanding of the functions and regulation of these kinases revealing potential common themes in the functions of RIPK1/3 and RIPK2. First, it became clear that RIPK1 and RIPK3, similarly to RIPK2, may function in the context of the innate immune responses by signaling downstream from the Toll-like receptor (TLR) family of PRRs (reviewed further in [3]). Furthermore, the IAP family E3 ubiquitin ligases, which have critical roles in innate immunity, can target both RIPK1/3 and RIPK2 suggesting further possibility of coordinate regulation. Second, while RIPK1/3 may primarily function as pro-death kinases, their activity *in vivo* leads to the robust induction of inflammation, reflecting the pro-inflammatory nature of necrotic cell death as well as still poorly understood cell death-independent inflammatory signaling by these kinases [3]. Third, both RIPK1 and RIPK2 have now emerged as critical mediators of intestinal homeostasis and drivers of intestinal inflammation. In the case of RIPK2, both loss-of-function of NOD2 and hyperactivation of this pathway may contribute to IBD (reviewed further in [2]). Similarly, loss of RIPK1 protein and hyperactivation of RIPK1 catalytic activity have both now been linked to the development of intestinal inflammation through the cell death-mediated loss of epithelial barrier function [4]. Moreover, perturbations in intestinal homeostasis in response to alterations in both RIPK1 and NOD2/RIPK2 have been linked to signaling from the commensal gut microbiota, which is an acknowledged causative agent in IBD and many other human inflammatory pathologies. In short, while the complex roles of RIPKs in pathologic and physiologic innate immune responses remain to be fully elucidated and are not meant to be thoroughly dissected in our letter, the current evidence raises an intriguing possibility that all three RIPKs may represent drug-targetable kinase activities contributing to an overlapping range of inflammatory pathologies associated with perturbations in the human microbiome.

Pertinently, three independent studies by our labs and the laboratory of Dr. Giulio Superti-Furga have suggested new opportunities for pan-RIPK targeting by small molecule inhibitors [5-7]. As a starting point, screening of clinical kinase inhibitors identified the third generation Bcr-Abl inhibitor ponatinib as a low nanomolar inhibitor of cellular necroptosis. Subsequent *in vitro* experiments revealed inhibition of recombinant RIPK1, RIPK2 and RIPK3 by this molecule [5-7]. Furthermore, Canning *et al.* reported the first crystal structure of RIPK2 with ponatinib facilitating future elaboration by medicinal chemistry [5]. Importantly, cellular analyses indicated that ponatinib inhibited NOD1/2, TLR4 and TNF-induced inflammation and cell death specifically through targeting RIPKs at low nanomolar concentrations. Ponatinib also efficiently inhibited RIPK1/3-mediated cell death and inflammation in a TNFα-induced shock model *in vivo* [7], suggesting that protective concentrations of this drug may be achievable with current dosing regiments [6]. Thus, while ponatinib has associated safety concerns, it represents a valuable tool to elucidate the potential of pan-RIPK inhibition in various models of inflammation linked to RIPK dysregulation as well as inflammation-driven tumorigenesis. In addition, derivatives of ponatinib show promise as selective RIPK inhibitors. In our work, we presented two directions for such elaboration targeting RIPK1. In one approach, we took advantage of the different binding pocket flexibility in RIPK1 *vs*. RIPK2/3 to develop RIPK1-selective analogs of ponatinib, termed CS series [7]. In the second approach, we turned to a fragment-based strategy, combining a hinge-binding fragment of ponatinib with a highly selective necrostatin-1 inhibitor of RIPK1. The resulting hybrid molecule, termed PN10, not only displayed low nanomolar activity in necroptosis assays, but also excellent selectivity towards RIPK1. As the binding modes of various newly reported RIPK inhibitors become available, these molecules may be similarly utilized to target RIPK2 and RIPK3. Other ponatinib scaffold modifications may also reveal common elements suitable for development of more selective pan-RIPK molecules. Overall, discovery of a clinically-relevant pan-RIPK inhibitor opens exciting new opportunities to elucidate common pathologic activities and to explore new therapeutic avenues targeting these kinases.
